# The Challenge of Cardiovascular Diseases and Diabetes to Public Health: A Study Based on Qualitative Systemic Approach

**DOI:** 10.1371/journal.pone.0132216

**Published:** 2015-07-14

**Authors:** Marilia Sá Carvalho, Claudia Medina Coeli, Dóra Chor, Rejane Sobrino Pinheiro, Maria de Jesus Mendes da Fonseca, Luiz Carlos de Sá Carvalho

**Affiliations:** 1 Scientific Computing Program, Oswaldo Cruz Foundation, Antiga Residência Oficial, Rio de Janeiro, RJ, Brazil; 2 Institute for Studies in Collective Health. Federal University of Rio de Janeiro, Rio de Janeiro, RJ, Brazil; 3 Department of Epidemiology, National School of Public Health, Oswaldo Cruz Foundation, Rio de Janeiro, RJ, Brazil; 4 Pontifical Catholic University of Rio de Janeiro, Rio de Janeiro, RJ, Brazil; Cinvestav-Merida, MEXICO

## Abstract

The most common modeling approaches to understanding incidence, prevalence and control of chronic diseases in populations, such as statistical regression models, are limited when it comes to dealing with the complexity of those problems. Those complex adaptive systems have characteristics such as emerging properties, self-organization and feedbacks, which structure the system stability and resistance to changes. Recently, system science approaches have been proposed to deal with the range, complexity, and multifactor nature of those public health problems. In this paper we applied a multilevel systemic approach to create an integrated, coherent, and increasingly precise conceptual framework, capable of aggregating different partial or specialized studies, based on the challenges of the Longitudinal Study of Adult Health – ELSA-Brasil. The failure to control blood pressure found in several of the study's subjects was discussed, based on the proposed model, analyzing different loops, time lags, and feedback that influence this outcome in a population with high educational level, with reasonably good health services access. We were able to identify the internal circularities and cycles that generate the system’s resistance to change. We believe that this study can contribute to propose some new possibilities of the research agenda and to the discussion of integrated actions in the field of public health.

## Introduction

The second half of the 1990s witnessed a debate between critics and defenders of Modern Epidemiology [1]. Criticism of the prevailing research model targeted excessive emphasis on the study of behaviors and other proximal factors [2], overlooking the importance of the physical and social context [3]; the methodological emphasis, with less attention to theory [4]; and the increasing distance to Public Health ultimate aims [5].

A decade later, there was clearly a renewed appreciation for the context in epidemiological research, as evidenced by the growth in publications [6–12] that approach empirical, theoretical, and methodological findings on the health effects of globalization, macro-social factors, place, and socioeconomic inequalities. Multilevel perspective [13] and life-course epidemiology [14] were adopted as a choice of research questions and the design of empirical studies. Statistical models incorporating structures of dependency between observations, especially multilevel models, became the standard analytical strategy in evaluations on the health effects of contextual factors, measured in the individual [15]. Nevertheless, counterfactual causality remained the predominant frame of reference, as in studies oriented towards the evaluation of risk factors at the individual level [16]. Statistical models are employed to isolate the effect of a factor, maintaining other potential confounding factors constant [17].

Recently, other methodological approaches, with systemic orientation [18–22], have been proposed to deal with the range, complexity, and multifactor nature of challenges associated with Public Health [23]. However, although such approaches tend to challenge the positivist perspective, compartmentalization, and reductionism [24,25], many authors have limited themselves to predominantly exploring some techniques, especially simulation [26], in addition to the usual research methods.

Scientific practice certainly faces enormous difficulties in questioning consecrated epistemological principles, methods, and criteria for validity, challenges that go far beyond the development of techniques and that run up against various cognitive and methodological limits [27]. The core of these challenges consists of dealing as plainly as possible with the complexity of the target phenomena [28]. Such complexity arises not only from the heterogeneous nature and the number of factors at play, but mainly from the interactions between them. The field of systems practices and studies includes philosophical, epistemological, methodological, scientific, and psychosocial aspects [29].

Considering only the systems methodological aspects, we can distinguish two major groups in the different watersheds of modern systems theories. On one hand, we have the “hard” methods that have their origin in cybernetics, operational research, and systems engineering [30], and which, to a certain extent, forgo breadth of scope in favor of rigorous mathematical formulation and the possibilities of computer simulation of dynamic systems [31] and agent-based models [32], among others. On the other hand, the “soft” systemic methods focus on creating dense and comprehensive qualitative models that value collective learning, creativity, and action [33].

Interestingly, although some authors view the “hard” systemic models as more rigorous, others warn against the imprecision in the models’ results and predictions: “*the model presented was a simple*, *abstract model that was not intended to be highly realistic or quantitatively calibrated to data”* [34]. This is curiously similar to some critiques of the so-called “soft” models.

Regardless of the approach, the systemic methods establish some strategies to deal with our natural cognitive limitations in face of large amounts of information. Among those are the conceptualization of models in layers. Although in mechanical systems this may be similar to the usual processes of Cartesian analysis [35], in psychosocial systems, where culture, information, and actors with resilience, subjectivity, and purpose are present, other, more abstract, criteria can command the conceptualization of subsystems and interactions in each layer. In addition, in the process and result of a systemic decomposition, the system’s “parts” are also (sub)systems, which both dynamically define and are defined by it. In this sense, our research focus on the conceptualization and configuration of the web of interactions, more than the parts per se. This is what Klir calls “systemhood”, as opposed to “thinghood” [36].

Among all chronic conditions, cardiovascular diseases are the leading source of disease burden in adults throughout the developing world and in the emerging economies [37], even if we consider the decrease in mortality related to the reduction in smoking and the availability of effective drugs for hypertension and hypercholesterolemia [38]. Meanwhile, the incidence of diabetes mellitus has increased “and preventive strategies–the battle to protect people from diabetes and its disabling, life-threatening complications is being lost” [39]. Despite having different risk factors and pathophysiological mechanisms, the two conditions share common causality [40], particularly for the more distal determinants, the “causes of the causes”. In addition, it is necessary to emphasize the role of the contemporary Western way of life, including dietary patterns, work relationships, leisure-time activities, and social and family networks.

As the Brazilian Ministry of Health recognized the problem in all its extent and severity, it supported the creation of a multicenter cohort study focused on these diseases, the Longitudinal Study of Adult Health–ELSA-Brasil [41]. ELSA-Brasil is a cohort study with 15,105 public employees from six universities and research institutions in Brazil. Its objective is to study cardiovascular diseases and diabetes (CVD&Diabetes) in relation to a wide range of biological, behavioral, environmental, occupational, and social factors.

However, notwithstanding the interest of the funder and the researchers in approaching the different disciplines and knowledge needed to create effective health policies for prevention at all levels, from primary to tertiary, such integration is difficult without an in-depth theoretical effort.

This paper presents a systemic model for CVD&Diabetes in the context of the ELSA-Brasil project, employing an original “soft” approach, whose theoretical foundations can be found in [42–45]. Our proposal is to create an integrated, coherent, and increasingly precise conceptual framework, capable of aggregating different studies, partial or specialized, allowing for the particularities, the various areas of knowledge, and the diverse modeling methods, all necessary for advancing research on the theme. The main purpose of this exercise is to build up a model capable of subsidizing strategic, administrative, and technical interventions based on a multilevel understanding of the systemic aspects associated with the phenomena under study. This aspect is particularly important, given the huge challenges for translating scientific discoveries into interventions, whether in public health or individual health [46]. For example, despite the efficacy of antihypertensive drugs, fewer than 70% of hypertensive individuals have their blood pressure under control. As an example of application, the model proposed in this study will be applied to the control of arterial hypertension.

### The Methodological Approach

The population health can be understood as a complex adaptive system [47], so we are essentially interested in discovering how a system maintains (as an organized whole) its properties, stability, and characteristics of evolution, under the influence of, or despite of multiple and continuous interactions with its environment [48]. Without this understanding, intervention efforts run the risk of frustration or undesired effects, since the system tends to “defend itself” globally and smartly. This dynamic stability of complex systems, whether containing conscious agents or not, can include quite profound adaptive changes, all the more powerful, the larger the system. If this were not so, the system’s very size would possibly have disorganized it, due to the natural entropy produced by the large amount and variety of internal and external interactions. Complex adaptive systems thus display some important characteristics: **emerging properties**, due to the composition and linkage between their pieces, and not to the individual characteristics of each piece; **self-organization**, a stable dynamic configuration to which it tends to return when momentarily disturbed; **feedbacks**, structuring the system’s self-organization processes and coevolution of its components; and **memory**, i.e., whereby the current organization also depends on the system’s historical conformation [49]. Paradoxically, systemic complexity does not produce just stability. The play of multiple interactions and circularities can also lead to the emergence of exponential trends capable of leading the system to unexpected and radical transformations, or even to its destruction. Resnicow & Page [50], dealing with human motivations in health-related behavior changes, call these breaks “quantum changes”, i.e., sudden and non-linear in origin.

Our approach is consistent with the “soft” thread of systemic thinking [31], and involves the collective and iterative creation of a complex, dense, and multidisciplinary vision, expressed in a diagram composed of symbols representing abstract subsystems, of various natures and scopes, interconnected by arrows that represent the relationships between them. The subsystems defined in this process can be either human, that is, with conscience and purpose, or physical/technical, from the perspective of actor-network theory [51]. The arrows are not just links indicating the existence of relationships. Their nature and content are described in depth, as the most important aspect for the understanding of the model.

The purposes of this and many other systemic modeling methods are: (i) to give meaning to and communicate complexity; (ii) to orient action strategies [52]; (iii) to raise new questions and themes for the pursuit of new knowledge integrated in a linked, consistent, and comprehensive vision.

Aware of the crucial importance of relationships in any systemic modeling, and of the fact that the relationships unfold and spread out more or less indefinitely, there is always valuation and volition in defining any system’s limits [19]. In our approach, the guiding criteria are the following: (i) in the description of the chosen “external” elements, one study just the relationships they maintain with the system under study not their mutual interactions; (ii) each internal subsystem of the system under study interacts significantly with at least two others, thereby establishing a cohesive and self-consistent model, capable of representing the system’s oneness and potential circularities.

The method thus emphasizes the description (as precise and rich as possible) of the **relationships**, each associated with a name and represented by an arrow connecting two subsystems. The overall limits of the study system are defined by a set of relationships that connect it to its environment systems, and not by a border, which would isolate it. Throughout the model’s creation, the relationships are specified with increasing clarity and precision, which also gradually conceptualizes and demarcates the subsystems and their position (internal or external) with increasing rigor.

Most relationships in such a model take shape as information (formal, informal, and even implicit), whose influence on the respective subsystems takes place through symbolic structures and contents, with no equivalent in quantitative expressions. Moreover, information does not impose on the connected subsystems any behavior or reaction. It just influences them. That's another strong and challenging characteristic of complexity.

There can be two types of relationships, directional and non-directional. In the former, one assumes that the subsystem at the origin takes the initiative of activating the relationship, in the form of an information flow. In the latter type, there is no precedence, i.e., the connected subsystems function in matched mode, mutually conditioned, without the need for an “initiative” by either of them. For example, a contract or agreement within a social group. There is also a situation in which one directional relationship activates another, in the opposite direction, and which constitutes its immediate answer or feedback. In this case, although they are actually two, these relationships are described together for greater clarity.

In our proposed approach (that we call “Direct Multilevel Method”), as we build the systemic model, the cognitive challenges of dealing with the problem’s complexity–scope, multidimensionality, and oneness–are tackled following some main principles: (i) subsystems and relationships are initially defined at a high level of abstraction and later decomposed and presented in detail; (ii) the degree of abstraction at each level is measured throughout the process by the amount of elements that emerge within it, taking into account the need to guarantee the consistency among the subsystems concepts; (iii) a relationship can only be decomposed by bifurcation when the subsystems it connects are decomposed in its subsystems; (iv) in the description of a specific subsystem or relationship, no reference is made to any other subsystem or relationship. In practice, in each level of decomposition no more then seven subsystems and 20 interactions should be included, to avoid the need for adjusting simultaneously an excessive number of concepts and texts.

The connection between the different descriptions is made exclusively by the diagram, which is thus the guiding element in the entire modeling. This guarantees the model’s consistency as a whole, while avoiding redundancies and contradictions among the texts that describes the various aspects of the model.

The description of any relationship, presented in tables, includes the following items, in addition to its **name** (that appears in the diagram): **definition**, **attributes**, and **examples/comments** (in general including references). At more detailed levels of the model, based on the decomposition presented here, one can also include the operational description, containing indications on the mechanisms and instruments that make the relationship effective in practice. When possible and relevant for the model, this operational description can include an indication of the relationship’s evolution over time. At more detailed levels of the model, it is also common to identify the events that trigger the relationship.

The “definition” explains and clarifies the relationship’s nature and semantic content. The “attributes” are the factors characterizing the behaviors associated with the interconnected subsystems according to the model’s objectives. Depending on the level of detail in the model, they can be expressed by text, variables, tables, or equations.

The “examples or comments” may include needs or opportunities for future research (including literature searches, additional data collection, methods etc.), bibliographic references, examples (for greater clarity), and, whenever they are not obvious, the semantic connections that may exist between the relationship that is being described and others, as a check on the model’s consistency. This column is dedicated basically to clarifying the model.

Importantly, from the systemic point of view, no relationship can be considered in itself as “causal” in the narrow or usual sense. A given subsystem’s behavior always results from **all** the relationships it maintains with other subsystems, many of which are feedbacks, together with the internal structure of its subsystems and relationships. It is a dynamic process with occasional mismatches. In this sense, the same model can superimpose all the way from qualitative descriptions to equations, from subtle influences to strong conditioning factors [53]. Therefore different categories of relationships can be classified according to their power of influence: rules, commands, requests, feedbacks, diverse forms of information or physical action, etc.

There is no “natural” criteria for the conceptualization of subsystems at each level of detail. They should only be different from each other [54]. Everything depends on the objectives of the exercise and some creativity. For example, the idea that “the larger contains the smaller”, whether in the physical or figurative sense–for example, the social level contains the individual, which contains the physiological, which contains the cellular–is useless in this approach. It is possible to mix “physical” with “abstract” subsystems, “small” with “large”, etc. Systems and subsystems are constructs. Their validity is due to the consistency of the descriptions of the relationships among all of them. Systemically, the conceptualization of a given subsystem is justified **by that of all the others**, provided that their interdependence is made explicit and defined as precisely as possible.

### The Model

**Fig 1:**
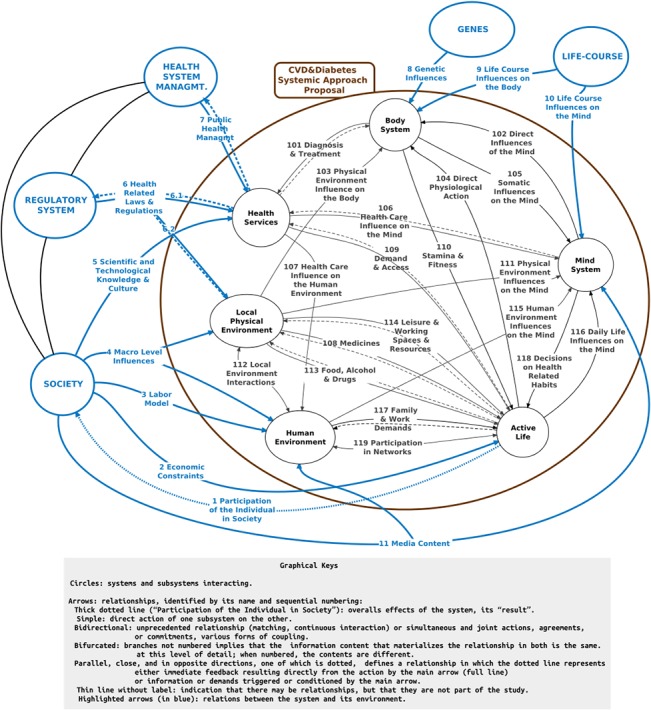
The System and its Environment.

The diagram in the Fig 1 expresses the whole system under study–“CVD&Diabetes Systemic Approach Proposal”–together with its external connections and environment systems, and its first level of “zooming”. Subsequent steps will give rise to other levels, necessarily integrated and consistent with the previous ones and with the knowledge and data already available or produced by other studies, collected gradually, under demand from the iterative modeling effort itself.

From the point of view of the objectives of the study, the system’s environment includes all aspects that conditions, influences, feeds, regulates, or receives the effects of this system. Five major environment systems were considered, conceptualized from the functional, abstract point of view. The first two–Genes and Life-Course–relate to and act directly upon the individual. The other three act on other subsystems. On Table 1 the main characteristics of the environment subsystems are described.

**Table 1:** Environment Systems.

System	Description
**Genes**	Individuals’ genetic characteristics.
**Life Course**	Individuals’ life course, including socioeconomic position, ranging from prior (family) conditions at birth until the beginning of the study, and on which there can be no direct action.
**Regulatory System**	All the legal instruments and bodies that regulate factors which can impact the target health aspects, except for executive or administrative functions. This system includes the functions of regulatory agencies, health professional boards and societies, and the various levels of the Ministry of Health and Secretariats of Health in their regulatory attributions. This system also includes the urban regulatory agencies acting in the areas of transportation, urban planning, security, education, and the environment. In practice, the influence of the “**Regulatory System**” on the “**Health Services**” subsystem takes place largely through the “**Health System Management**”, and not directly. However, given the study’s focus, we can assume that this influence acts directly on the “**Health Services**” subsystem, without sacrificing the model’s rigor or intelligibility.
**Health System Management**	All the functions that directly manage or direct resources and services that impact health. This environment system includes insurance companies, as well as federal, state, and municipal management bodies (acting mostly on public services).
**Society**	The other general aspects of the social environment in which people live–cultural, organizational, social, political, economic, and institutional–that can exert an overall influence on individuals’ psychological and social lives, as well as the subsystems with which they interact in their active social, physical, and psychological lives. In this study, Society also represents the environment system that receives the effects or end “products” of the study system.

Internally, the study system is depicted as containing the following subsystems, which interrelate dynamically and constantly, thereby affecting each other (Table 2).

**Table 2:** First Level Subsystems.

Subsystem	Description
**Body System**	The individual’s functioning body in relation to the target attributes, including measurable indicators (glycemic index, BP, BMI, ECG, etc.), self-reported diseases and incident diseases, etc. The aim is to understand the behavior of these attributes in light of the system as a whole.
**Mind System**	All the psychological aspects that can directly or indirectly affect the other subsystems, including more or less healthy choices. For example: mood, expectations, hopes, and dreams, as well as beliefs, values, habits and addictions. In brief, the mental and behavioral patterns that in general are resistant to change.
**Active Life**	Individual daily activities: eating, shopping, physical exercise, transportation, housing, rest, leisure, and cultural, social, religious, and political activities.
**Local Physical Environment**	All the physical aspects, including the neighborhood–transportation, pollution, temperature, sanitation and security conditions, availability and types of commerce, health services and pharmacies, places for exercising–and the work-related physical environment–time and comfort in commuting, workplace, ergonomic aspects, and food quality and accessibility.
**Human Environment**	All the social milieu: family, neighborhood, work environment, friends, social media. These different networks can overlap.
**Health Services**	Includes both public and private services, their operational characteristics, location, organization, and functioning, technical resources, availability.

Tables 3 and 4 describe the model’s external and internal relationships, identified by their numbers.

**Table 3:** External Relationships.

R	Title	Definition	Attributes	Examples, Comments, References
1	Participation of the Individual in Society	The way individuals participate in society (work, social and family activities, etc.), considering their current and past cardiovascular and metabolic status.	Physical and mental limitations on social activities (work etc.). Burden on the health system, work, study, social security, family, social environment, and public infrastructure. DALYs (years of life lost to disease, disability, or early death).	Overall participation: tax-paying, assiduity, productivity, participation in social movements, support for friends, participation in political parties [55–57]In Brazil, individuals with severe heart or kidney disease are exempt from paying income tax after they retire [58]. Medical limitations: sick leaves, early retirement [59], low productivity, overburden on family [60], disability or physical and mental limitations [61].
2	Economic Constraints	Wage restraint and legal obligations.	Family income, tax burden, social security payments and complementary expenditures, other expenditures on essential services not included above.	Wages, taxes, health plans, and government and private social security [62,63]
3	Labor Model	Formal or implicit principles, customs, regulations, and rules that influence values, working contracts and conditions.	Expected work pace, quality, and productivity. General values (real or publicized) of contemporary work: adherence to the organization, autonomy, pro-activity, availability, individualism, competitiveness/collaboration, continuous improvement, lack of privacy. Job stability. Labor rights and duties.	Information technology leading to decentralization, individualization, overlapping, and interference between time dedicated to work and private life, vacation. Retirement plans and age at retirement. Organization of work space and work at home [64,65]
4	Macrolevel Influences	General economic, urban, and social policies that influence and condition both the local physical environment and the human environment.	General state of the economy and economic measures. General laws and regulations affecting everyday life, including mobility, leisure, neighborhood, and public security. Political expectations and atmosphere.	Education, security, sanitation, green areas. Interest rate policies, subsidies, and other general economic aspects. Unemployment, wages, income inequality. Climate and environmental change [66–71].
5	Technical and Scientific Knowledge and Culture	Medical and scientific knowledge and respective technologies and culture, attitudes, beliefs, and values.	Nature of technical training: specialist versus generalist, scientific training, training for relationship doctor-patient and for interpersonal communication, integration with technological resources. Quality of specific and humanist training. Attitudes, beliefs, and values, including job hierarchy. Understanding of the multidisciplinary need for patients treatment processes and teamwork.	Incorporation of evidence in health care practice, indication of lab tests versus clinical examination, physician-patient relationship, appreciation of curative medicine over prevention, specialization, job hierarchy [18,72].
6.1	Health related Laws and Regulations (main direction)	Resolutions, regulations, decrees, and laws related to public and private Health Services.	Budget (public sector)–values, priorities, and allocation. Organizational rules–staff distribution, location, and attributions, referral and counter-referral, administrative and decision-making systems, rules for hiring services, equipment, and supplies. Rules for adjusting fees and minimum operational requirements (private sector).	Regulation of health services spatial distribution, health care practices, health professionals’ work, list of procedures under private health plans, price control in health plans. “Farmácia Popular”[73]. Specific health programs (hypertension and diabetes). Government spending on health as a share of GDP. Budget allocation [74–82].
6.1	Health related Laws and Regulations (*opposite direction*, *dotted)*	Demands for regulation originating from health services; data (feedback) from health information systems for diagnosing, evaluating, and monitoring regulatory actions (for potential adjustments).	Nature and degree of political power in private demands for freedom of prices and contractual conditions. Various demands for resources and adjustments (public sector). Nature, periodicity, and quality of control information (feedback).	Private insurance companies’ lobby [83], health-related legal suits, and access to healthcare [84].
6.2	Health Related Environment Regulation (main direction)	Resolutions, regulations, decrees, and laws that directly impact the physical environment.	Themes (pollution, security, transportation, sanitation, municipal ordinances), extension, focus and scope of environmental resolutions.	Ban on smoking in public places; regulation of salt consumption, preservatives, trans and saturated fats; regulation of foods sold in schools; creation of green spaces, street lighting [79,85–88].
6.2	Health Related Environment Regulation (*opposite direction*, *dotted*)	Local demands by a city or neighborhood, and general demands, including structured or unstructured information on the surrounding physical environment.	Quantity and nature of demands and feedback information.	Community participation in urban development, websites for lodging complaints [89,90].
7	Public Health Management (main direction)	Decisions, human resources, supplies, and infrastructure, as well as direct administrative and operational actions on Health Services.	Nature, quantity, and quality of human resources and infrastructure, including the permitted degree of flexibility and autonomy; nature, frequency, timeliness, clarity, feasibility, and pertinence of governance orientations and decisions.	Allocation of human and physical resources (consultation rooms, health care establishments, equipment) [91,92]; public-private relations in health care administration [93]; direct information and orientation for services and operational and administrative information systems [94]; permits and interdictions, certification [95].
7	Public Health Management (*opposite direction*, *dotted*)	Demands for decisions, resources, and actions; information on health services operation	Nature, pace, style, and quality of demands, including their realism; information (informal or through formal information systems) on the operation, performance, demands, and problems.	The right to health, health activism [96].
8	Genetic Influences	Genetic influences associated with propensity to CVD&Diabetes	Physiological parameters that can identify these tendencies.	Research on genetic influences of CVD&Diabetes point to wide variability. Components of family history [97–101].
9	Life Course Influences on the Body	Influences of previous life course on the study’s target aspects.	Individual risk or protective factors originating in the previous way and experiences of life.	Eating patterns, physical activity, smoking, alcohol consumption, intrauterine growth restriction, previous diseases (infections); characteristics of the physical and social environment to which the individual has belonged over the course of life, and which are incorporated biologically (embodiment)[102,103].
10	Life Course Influences on the Mind	Influences of life course on current Mind System as related to cardiovascular and metabolic health.	Stress/anxiety–chronicity and duration–and resilience; perception of life: optimistic/pessimistic, active/passive; spirituality and religion; responsibility for one’s own life; schooling, culture, and general information.	Self-awareness, understanding, and disposition for habit changes; constant financial and family problems; habits and vices; schooling and general knowledge; traumas; previous psychotherapies [104–106].
11	Media Content	Set of information and symbols that affect the overall state of spirit, including overall tendencies, expectations, and hopes concerning the current situation.	Nature of advertising, mass media, and cultural offerings on life ideals: status, economic standard, lifestyle. Nature of the real or imaginary political, social, and economic atmosphere published or broadcast by the media.	Misinformation on access to health services, quality, risks [107]; advertisements for cigarettes, food products, drugs, alcohol, behaviors [108]. News that stimulates a perception of fear (violence, diseases, deaths); process of shaping public opinion, feelings, wishes, and needs, with local and general characteristics, affecting individual and group self-esteem [109–112].

**Table 4:** Internal relationships

R	Title	Definition	Attributes	Examples, Comments, References
101	Diagnosis and Treatment	Diagnostic approach–patient history, clinical examinations, imaging and lab tests, and respective direct therapeutic management when it exists. This relationship does **not** include prescriptions when they are given to patients to follow later on their own.	Degree of adherence to protocols/ guidelines; integrated individual approach versus medical specialties; quality of physical environment in the health care services and humane care; duration and waiting time for examination, tests, and treatment; repetition of tests and interventions; test processes or direct therapeutic action.	Efficacy and effectiveness studies; harm and near-miss [113–116].
102	Direct Influences of the Mind	Mechanisms by which psychological status directly affects physical health (not including indirect mechanisms, via behaviors).	Psychological status evaluated by scales (stress, quality of life, satisfaction) and association with incidence and aggravation of CVDs and diabetes.	Since psychological status can also indirectly affect physical health through the sequence 'R118—changes in Active Life–R104', caution is necessary in the determination of this direct correlation, if it actually does exist [117–119].
103	Physical Environment Influence on the Body	Mechanisms by which the local physical environment directly affects physical health.	Climate–temperature, relative humidity, precipitation, radiation; indoor heating and air conditioning; air, water, and sound pollution; smoking (passive); type and quality of transportation.	Impact of climate and pollution on mortality from myocardial infarction and stroke, indicating aggravation of CVD (but not incidence) [120]. Noise levels possibly relate to physical health by increasing stress (R111). Environmental components can be measured directly with instruments or indirectly through questions about the environment. Modeling can include time series, spatial studies, or hierarchical regression models [121,122].
104	Direct Physiological Action	Direct physiological influence of habits and behaviors on physical health.	Smoking and alcohol consumption; diet: sugar, salt, fats, fruits, and vegetables, quantity and quality; physical exercise: type and intensity; sleep: pattern and quality; time and effort spent commuting from home to work and in other daily activities; time and quality of leisure.	Studies associating behaviors and respective scales and ways of measuring eating habits, physical activity, smoking, use of time, among others, are more frequent in the literature, focused on evaluating the association between dozens of individual behaviors and their outcomes [123,124].
105	Somatic Influences on the Mind	Direct influences of physical health status on the “Mind System”.	Physical factors that directly affect psychological status, including the study’s target diseases (CVDs and diabetes); diseases that cause constant pain or limit activities; diseases that lead to depressive states and lack of disposition or anxiety; associations between these states and the different factors that characterize the “Mind System” (see R102).	As in R102, various studies have evaluated the association between CVD&Diabetes and mental health [125,126].
106	Health Care Influence on the Mind (*main direction*)	Prescriptions, orientation, information on health problems and their implications; implicit messages on behaviors (diet, alcohol consumption); reminders to comply with recommendations and orientation; information, warnings, and orientation from health services, including outside the health service setting, to reinforce prescriptions.	Quality of physician-patient relationship in the sense of obtaining relevant information; quality, adequacy (protocols), and clarity of prescriptions, considering individuals within their socioeconomic and cultural context.	Direct evaluation of the information component [127]; indirect evaluation through verification of compliance with recommendations [128]. Formal information–prescriptions, recommendations, and orientation on tests and use of medicines–is complemented by non-verbal information that makes the individual more or less prone to following the formal recommendations, which may even contradict them. For example, a health professional who smokes, but who recommends that the patient quit smoking [129]
106	Health Care Influence on the Mind (*opposite direction*, *dotted*)	Information on life situation, complaints and facts associated or unassociated with CVDs and diabetes; demands for care; immediate responses (explicit or subtle) to the process of care.	Form, quality, and content of information, complaints, demands, and reports by patients; patient’s reaction to the information received.	Comprehension of medical recommendations in different socio-cultural contexts [130]; physician-patient communication [131].
107	Health Care Influence on the Human Environment	Programmed intentional and objective information or involuntary information affecting collective behaviors and practices (family and social networks), via self-help groups, waiting room, informal indications and evaluations.	Programmed or unplanned means for transmitting information; content and nature of information (positive, negative, derogatory, endorsements, etc.).	This relationship can be evaluated through intervention studies, always considering groups rather than single individuals. Social networks have an important impact on this relationship [132,133].
108	Medicines	Acquisition of drugs and other therapeutic products	Drug prices in relation to living standard; ease of access; variants–generics, substitutes recommended by the pharmacist or others and sources of information; adequacy or contraindication, whether according to the prescription or via self-medication; multiple diseases and drug-drug interactions; adequate use of medicines.	Can be studied at the individual level, verifying whether the drug was obtained, and at the health service level, verifying availability[134].
109	Demand and Access	Individual search for and access to Health Services.	Time between stages of care: demand for and scheduling of appointments, tests, results, and intervention; level of information and knowledge after contact with the health service.	Various approaches to study access to and use of health services [135] and various theoretical models proposed to study aspects of access to the health system at its multiple levels [136–138]. Survival models seeking variables associated with each of these times, including individual and health service aspects (multilevel).
110	Stamina and Fitness	Individual physical resources, potentialities, and autonomy that allow different types of action.	Resilience, chronic fatigue, functional capacity; scales of the Activities of Daily Living type	Various aspects of fitness can be tested, ranging from stress tests to questionnaires and scales. The concept of frailty, used in studies of the elderly, evaluates loss of vitality [139,140].
111	Physical Environment Influences on the Mind	Direct influences on Mind System and health from factors that are not controllable by the individual, associated with the local physical environment.	Noise, extreme weather conditions, lack of sunlight (more common in countries with temperate climate), filth, disorder, poor maintenance and appearance, pollution, inadequate artificial lighting, crowding, lack of privacy; relationship between each of these attributes (or sets of attributes) and the psychological states in the Mind System subsystem.	Association between local physical environment and aspects of psychological health, generally mediated by perception of the local environment [134]. New methodologies for objective measurement of the local environment have been proposed [141].
112	Local Environment Interactions	Ways of mutual conditioning between the physical and human environments, or the types of interaction by which the local physical environment affects and is affected by the local human environment, and the two subsystems adjusted to and are constructed by each other mutually and simultaneously.	Types of social structure: organizations–churches, associations, clubs, LAN houses; observable informal structures–youth groups, illicit drug outlets, alcohol and drug consumption; sports, cultural activities; human atmosphere of the environment (leadership, respect, values, level of aggressiveness or cooperation).	Poorly conserved environment, with broken equipment, can produce in the human environment states of discouragement, pessimism, and aggressiveness that aggravate the state of the equipment, increase the filth (reinforcing the state of things). Inversely, a positive and collaborative human environment can lead to better, permanent conservation of the physical environment [142].
113	Food, Alcohol and Drugs	Acquisition of all types of food and legal or illegal drugs.	Availability and accessibility (including economical), physical and cultural.	Ease of access to junk food, cigarettes, drugs, and alcoholic beverages, or fruits and vegetables [143].
114	Leisure and Working Spaces and Resources	Supply and demand of urban equipment in the neighborhood, for commuting, and for professional activities that allows and conditions daily activities.	Availability, costs, and types of resources for leisure, work, and regular physical activities; conditions of urban mobility; time spent in various daily activities; effective use of different resources.	The components of urban commuting, leisure areas, equipment in the neighborhood for exercising, and available time [138].
115	Human Environment Influences on the Mind	Direct influences of factors associated with family, coworkers, neighborhood, and social networks.	Support and forms of social contact, characterization of conflicts and pressures, group pessimism or fear; configuration of networks of influence (including leadership), pressures from the work environment, discrimination, symbolic influence of food, pressures for alcohol consumption, inadequate foods, drugs, and alcohol and/or behavior changes (smoking cessation, exercise, diet); family-work conflicts, conflicting pressures from the local human environment.	Studies of social support and other psychosocial factors and relationship to CVDs and diabetes [144–146].
116	Daily Life Influences on Mind	The ways by which individual daily activities, experiences, and actions directly affect Mind System, including maintaining or modifying the mental status quo (habits).	Physical exercise and leisure-time activities; psychotherapy and physical therapy; cultural, spiritual, or religious activities; perceived pleasure from foods, alcohol, and other substances; pleasure obtained from social relations; time in the consolidation of habits that affect the target aspects and characteristics of attempts to change.	Each constant and repetitive activity tends to perpetuate itself due to the habit itself (otherwise it would not have become constant and repetitive). On the other hand, some activities, like those that influence evolution and change (studies, therapies, religion, change in network of friends or jobs, etc.) may foster changes over time [147,148]. This relationship does not include the activities’ physiological influences (high-calorie foods, smoking, alcohol, exercise, etc.) described in R104.
117	Family and Work Demands	Demands and pressures that influence the types, frequency, and intensity of individual activities.	Nature, time, and volume of work; demand and control over activities, job position and responsibility, schedules, invasion of personal space; available time for non-work activities; time spent on dreary or unwanted activities; constant use and availability of cell phones and time used accessing the Internet.	Studies associating workplace stress scales and CVDs and diabetes; division of time between work, family, and rest or leisure-time activities; availability and quality of family life [149,150].
118	Decisions on Health-Related Behaviors	Choices and decisions that trigger and orient all the behaviors related directly or indirectly to health, whether positive or negative, conscious or unconscious, regular or irregular, permanent or sporadic, well-informed or poorly informed.	Personal history of decisions, whether consistent, evolving (learning), or failed; degree of perception, information, awareness, impulsiveness, and coherence of usual decisions (including challenges to sustain more serious decisions); areas of behavior: smoking, sleeping more or less, taking on more jobs, attending stressful environments or activities, healthy eating, sedentary lifestyle, social and spiritual life, leisure, choice of groups.	Motivational studies apply to this relationship, which is fundamental to the entire process. The importance of the relationship is not reflected clearly in therapeutic actions by health professionals, considering that drugs are the “cause” of therapeutic successes, while failures are viewed as the individual’s problem [151].
119	Participation in Networks	Influences of participation in different social networks.	Types of networks; frequency, dependence, and time spent participating; individuals’ location in their networks’ structure and topology, indicating their degree of autonomy and their influence on them (which simultaneously compromises maintaining oneself active).	Techniques and tools for studying networks [152]; specific interest groups (e.g., weight-watchers, mountaineering clubs) or general groups (family, work groups, neighbors) and their impact on behaviors associated with CVDs and diabetes [153–155].

### Overall Comments on the Model

The highlighted arrows in Fig 1 indicate the system’s relationships to its environment. A highly important overall observation is that the system’s main feedback to the environment (R1 –Participation of the Individual in Society) has a long time lag in relation to its inputs as a whole (R2, R3, R4, R5, R6, R7, and R11). The inputs-output connection is not evident and hardly intuitive. Therefore, the relationship R1 provides actually very little feedback. Its quite hard for individuals, policy-makers, politicians, and businessmen to perceive, with some clarity, the influence of their actions on the system. This limitation, characteristic of any complex phenomenon, is particularly crucial considering the food and resembling industries, which are part of the “Society” system and act through the relationships R2, R3, R4, R5, and R11. Consider, for example, an action driven by business interests that are contrary to the population’s health [156]. It's quite hard to elicit or question such interests, thus hindering trustful negotiations between government and businesses or the adoption of comprehensive and long-term health policies.

On the other hand, some feedbacks can be planned and more oriented. These are represented in the diagram by the dotted arrows for R6 and R7 and serve to affect directly the behavior of the Health System Management and the Regulatory System. However, their response on the system CVD&Diabetes is not direct or immediate. It so happens because part of this response is exerted indirectly on the Society itself, through general laws and regulations. A few successful examples are the ban on cigarette advertisements, negotiations with the industry to reduce sodium in industrialized food products, some direct economical measures and incentives.

### High Proportion of Non Controlled Hypertension: What the Model Can Tell Us?

In the context of the ELSA-Brasil study, 34% of participants responded that a physician had already told them that they had hypertension, and 29% were taking medication to control their blood pressure. Among those who knew they had hypertension and were taking antihypertensive drugs, 31% did not have their blood pressure controlled, corroborating data from various studies in other populations [157,158]. The percentage of blood pressure control was higher in women. More schooling was associated with better blood pressure control among users of antihypertensive medication, with a clear gradient between those with the least schooling (43% without their blood pressure controlled) and those with graduate degrees (only 22%).

From the systemic point of view, the question is: considering the existence of effective antihypertensive drugs, in an educated population and stable employment, what could explain such high proportions of failure to control blood pressure?

**Fig 2:**
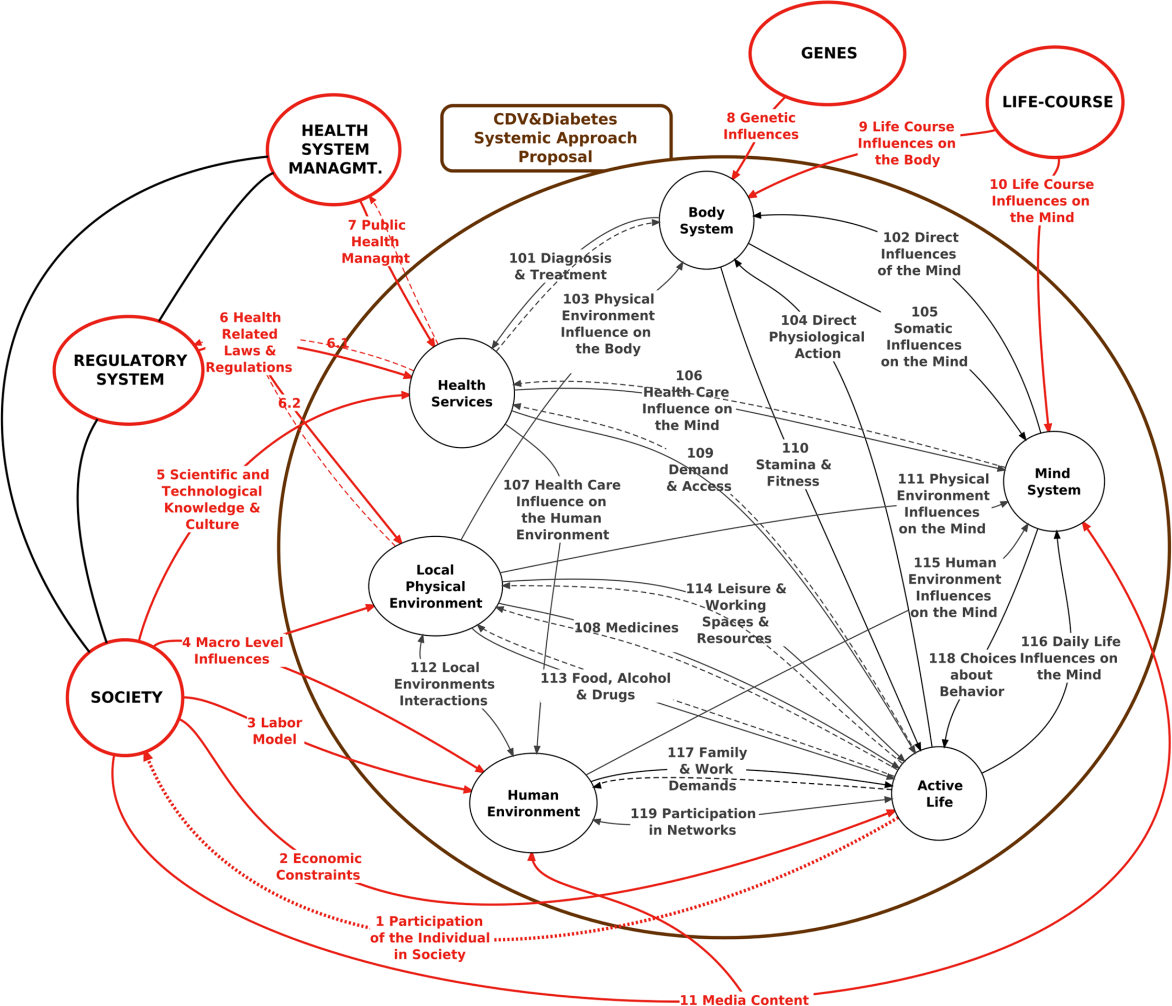
Direct Influences on the Body System.

Hypertension is a set of attributes of the Body System, and, in our model, results from the direct and **combined** influences of the relationships R8, R9, R102, R103, and R104 (highlighted in Fig 2), compensated for or regulated by homeostatic physiological mechanisms, internal to the Body System (that will be studied in the model’s future and more in-depth development).

The Genetic Influences (R8) and the Life Course factors (R9), resulting from personal experiences accumulated since intrauterine life, are not modifiable and influence the primary individual conditions of propensity to hypertension [159–161]. In addition to these, three relationships act independently on hypertension: Physical Environment Influence on the Body (R103), Direct Physiological Action (R104), and Direct Influences of the Mind (R102) (the last one will be discussed later on). The individual's active influence on the Body System's blood pressure (BP) occurs predominantly through the relationship R104, which comes from the Active Life subsystem, where the specific actual healthcare or health damaging actions can take place, including taking medication, plus lifestyle. If, even when therapeutic measures are taken, the person’s BP is not maintained within a “healthy” range, we have an example of “cybernetic causality” [162]: the situation under study is due to the **absence** or **weakness** of one or more **corrective cycles** rather than to “causal” factors. In other words, to reach and maintain adequate blood pressure levels requires the presence of continuous compensatory systemic cycles, one of which is the treatment itself.

**Fig 3:**
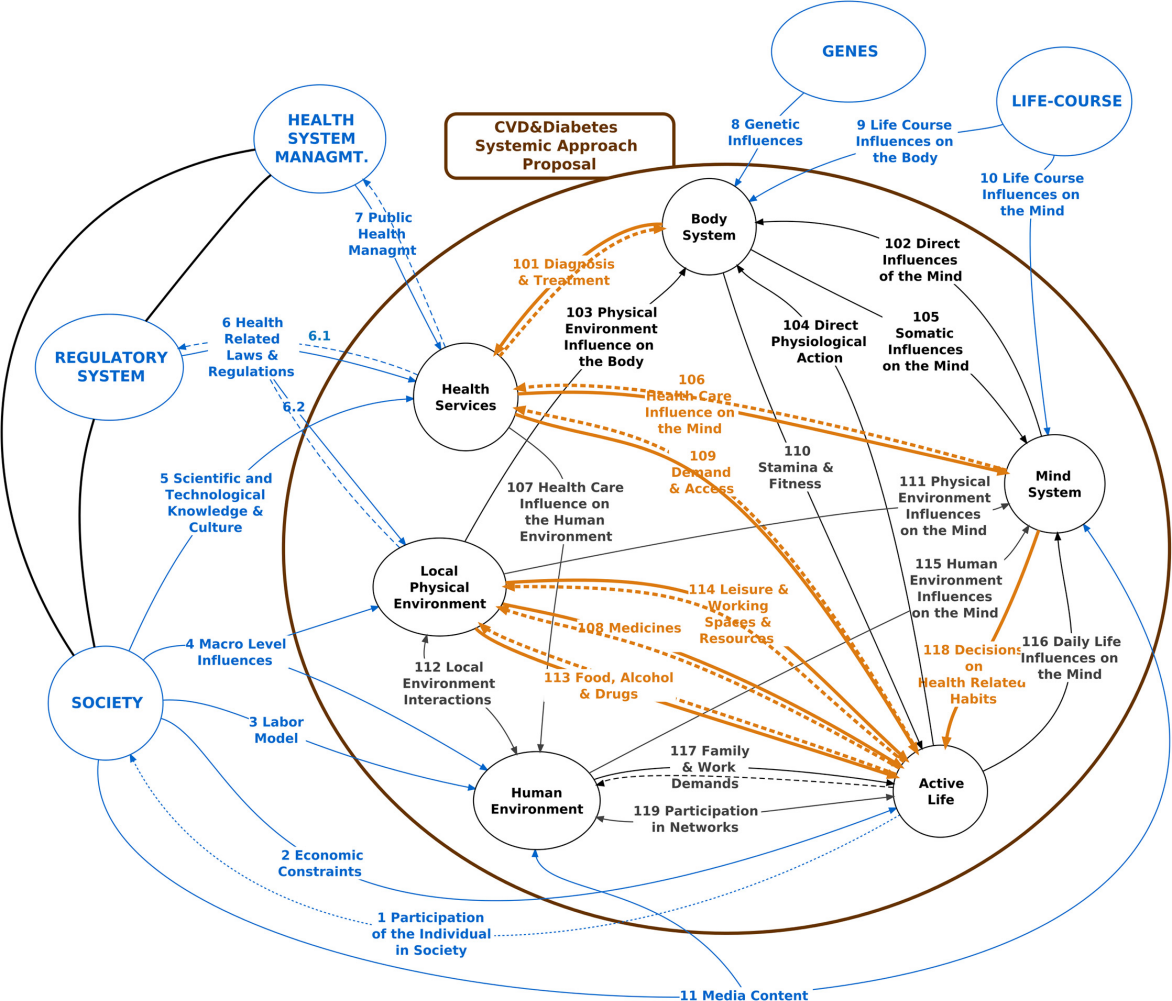
Cycle 1 –Restoration and Maintenance of Adequate BP Levels Through Lifestyle and Treatment.

**Fig 4:**
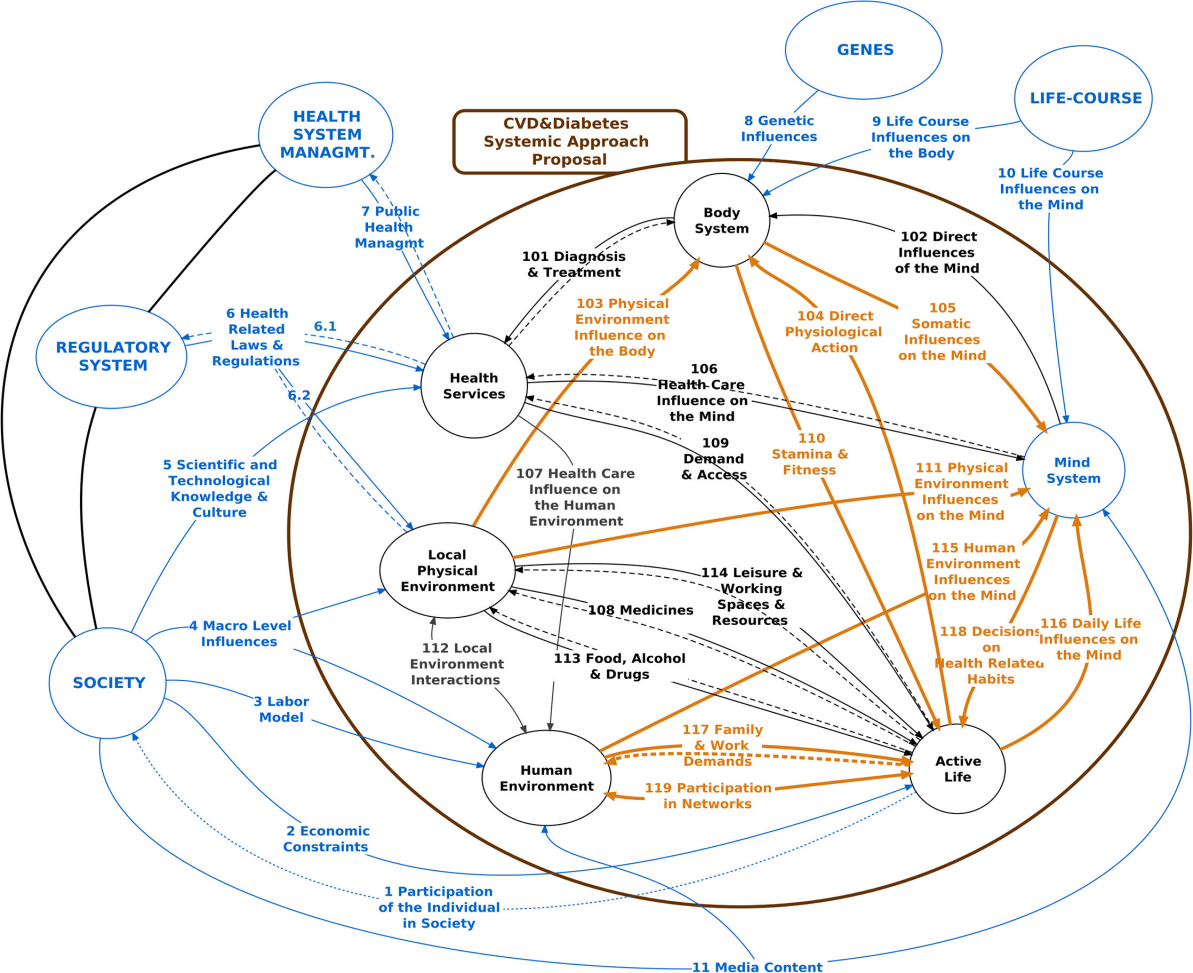
Cycle 2 –Main Constraints on Behaviors Associated to BP.

**Fig 5:**
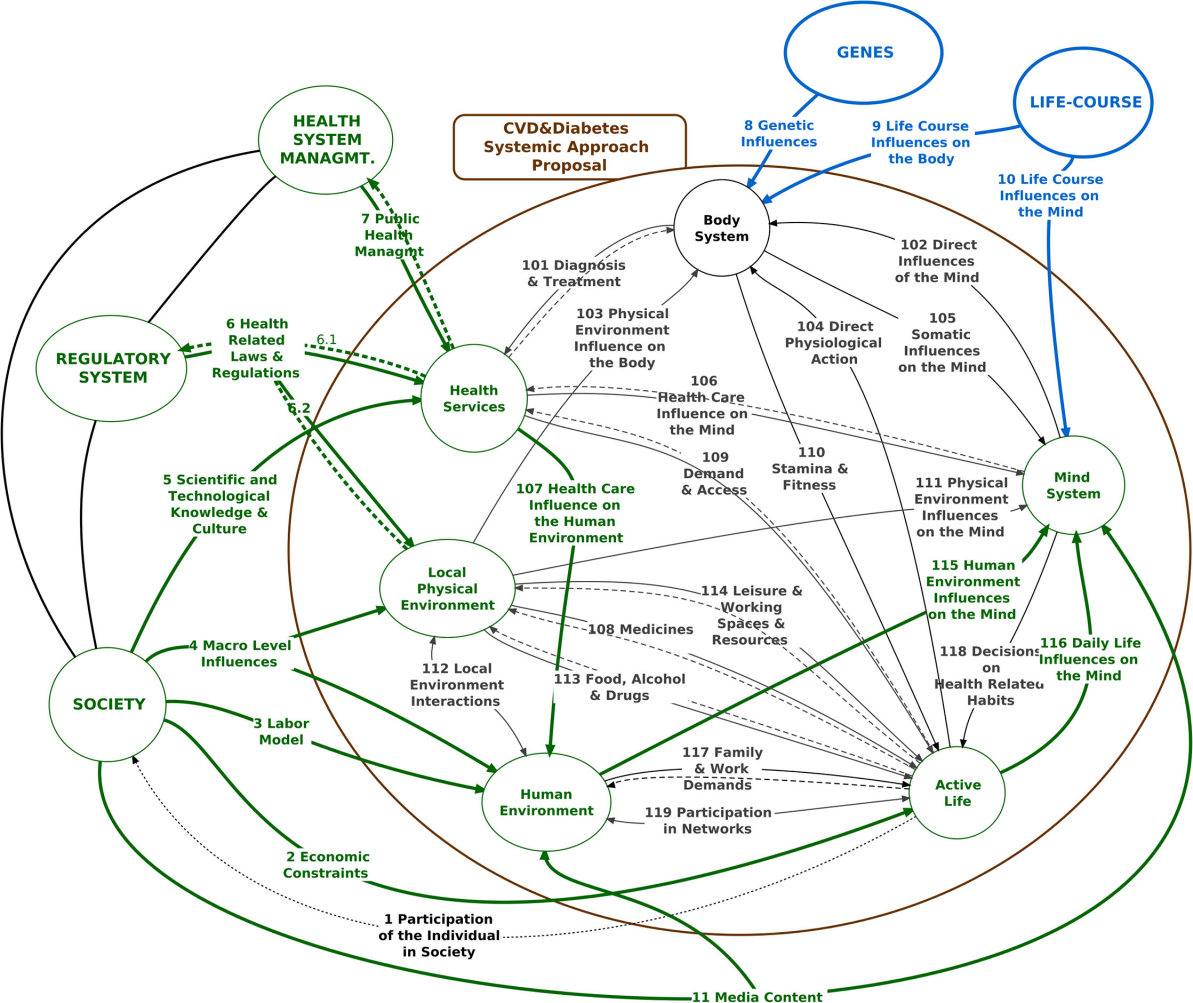
Influences of the Context on the Cycles.

We analyzed three cycles of the model (Figs 3–5), highlighting in different colors the main paths of each cycle.

Cycle 1 shows the main actions for the detection, treatment, and follow-up of hypertension. Since this is a chronic disease, its control requires regularity and constancy. Let's discuss a first sub-cycle, represented by the R109-R101-R106-R118-R109 loop, witch is aimed to the **regularity** of the follow-up. Under ideal conditions, once the individual access the health service (R109), a clinical examination is performed and the BP is measured (R101). Relationship R106, which follows, includes information from the patient to the physician–reported symptoms, results of previous consultations, tests, and prescriptions, report of habitual behaviors, among which physical exercise and diet, family history, and other important data for BP control–and from the physician to the patient–prescription of medications, clarifications and orientation on other aspects of the disease, and recommended behaviors. The next relationships (R118-R109) closes the loop, maintaining the follow-up and control routine as well occasional adjustments in the treatment, which are meant to guarantee BP stabilization at adequate levels. When this fails to happen, the usual hypotheses for investigation in this sub-cycle are specific failures located in one or more of its interactions, including in R101 and R106, for example, inadequate prescriptions [163].

From the systemic point of view, however, it is important to emphasize some nonlinear and barely evident phenomena, such as the **natural self-reinforcement** of a **regular** or **irregular** behavior. Supposing, for example, that the power of the relationship R106 to counteract the psychological patterns and other relationships (see Cycle 2 described later) is insufficient to provide (in the Mind System), the **given** and **stable** attitudes that lead to the decision (R118) to return repeatedly and regularly to the health service. In this case, the irregularity or prolonged interruption of follow-up (R109) may, for example, prevent some necessary prescription change or may jeopardize the physician-patient relationship (R106), further reducing the likelihood that the patient will return to the health service. And vice versa: if the information and influences from the care (R106) promote, through adequate awareness-raising in the Mind System, the decision (R118) to control BP, taking the proper and regular measures (via R109) to return to the health service for follow-up, this will tend to reinforce the quality and power of the relationship R106 at the next appointment, thereby creating a virtuous circle. With this example, we intend to highlight one of the most important qualities of systemic thinking: in most cases, it does not suffice to investigate specific failures in the different parts of a specific sub-cycle. It is also necessary to consider the **circularities that propagate and reinforce these failures**.

The second sub-cycle in Fig 3 is centered on the Active Life subsystem, namely on voluntary individual actions. Under the influence of R118, the relationships R108 (acquisition of Medicines), R113 (acquisition of Food, Alcohol and Drugs), and R114 (adequate lifestyle through access to Leisure & Working Spaces and Resources) act on the subsystem Active Life that, in its turn, triggers a Direct Physiological Action (R104) on the Body System.

The relation R108 indicates the acquisition of drugs prescribed to control BP. Although Brazil’s Unified Health System (SUS) supplies various antihypertensive drugs free of cost, their supply or acquisition may not be regular for various reasons, including geographic accessibility or lack of the drug in a given health service. The actions in the relation R114 are the search for and use of resources for physical exercise, an important aid in controlling hypertension. These actions are conditioned by the existing infrastructure and suffer important restrictions from the time expended per day, for example, on work, transportation, and household chores, among others. The relation R113 completes the sub-cycle and includes access to and acquisition of healthy foods, for example, or of tobacco and alcohol, the latter known to be associated with high blood pressure [164].

Keeping in mind that the actual functioning of this entire process involves the subsystems Mind System and Active Life, its main weakness, from the individual’s point of view, is the lack of **clear, direct,** and **short-term feedbacks** that orient adjustments in behaviors to favor the control of hypertension, which is generally asymptomatic. That is, modification of the behaviors in R108, R113, and R114 –acquisition and consumption of adequate foods and drugs and physical exercise–and the search for regular medical follow-up (R109) do not bring immediate benefits.

A final remark about the Cycle 1 is that the information about recommended prescriptions and actions (R106) occurs at a predominantly **rational** level, generally weaker than the level of direct perceptions, wishes, habits, and emotional decision-making mechanisms, which define R118 in the first place, and whose characteristics may led to potentially harmful behaviors through R108, R113, and R114 [165].

The Cycle 2 (highlighted in Fig 4)–underlying behaviors related to hypertension–is even more complex, involving R103, R104, R105, R110, R111, R115, R116, R118, R117, and R119. It mainly considers the influences on the individual sphere (Mind System and Active Life), with various relationships that connect to those discussed in Cycle 1. Likewise, **it lacks any observable feedbacks**, showing that without **conscious, consistent, and active participation by the subject**, medical prescriptions and interventions alone do not suffice to maintain adequate blood pressure levels [166].

Various hypotheses should be considered. The presence of comorbidities such as diabetes, a relatively frequent condition in individuals with hypertension, brings an additional set of drugs and routines that can affect Mind System (R105) and interact with disposition to control blood pressure (R118). The Local Physical Environment–lighting, noise, pollution, contact with nature–can have a direct effect on BP (through R103 [167] and R111 [168]), affecting behavior-related decisions (R118) to control BP. Social networks and the individual’s family not only influence adherence to healthy behaviors (R118) but can also play a stressful role (R115) directly associated with the control of hypertension [169].

Three important **closed reinforcement sub-cycles** of behaviors in Active Life should also be considered. One of them, inertia or **force of habit**, corresponds to the short sub-cycle R116-R118-R116. The other is R117-R119. They involve aspects that directly affect Active Life: pressures and demands on time and attention (R117), potentially reducing the availability for activities that balance BP; the direct and self-reinforcing influence in and from social networks (R119), leading for example to eating and drinking inadequately and to sedentary lifestyle (or, on the contrary, to adopt healthy habits). The third sub-cycle (R110-R104) brings a positive feedback, involving direct physiological action of Active Life on the Body System and the corresponding response as Stamina and Fitness (R110). For example, dedicating more time to physical exercise increases vitality. On the other hand, obesity decreases the capacity to practice exercise. The latter limits the possibilities for favorable changes in Active Life, producing a circularity that maintains hypertension.

Highlighted arrows in Fig 5 show the contextual influences on the cycles previously studied, the analysis of which can orient safer and more effective interventions. The most obvious ones are on the Health System Management and on the Regulatory System. However, although with a longer time lag, educational, political and legal interventions on the Society system can have profound and possibly more lasting impacts.

The Society system influences individuals’ Mind System through Media Content (R11), establishing values and stimulating wishes, in this case food and beverage advertisements, with a short-term impact. This influence (R11) also occurs on the local Human Environment, which in turn, as we saw in Cycle 2 (R115), affects Mind System, reinforcing the direct influence on the individual. Society also sets economic standards (R2) and a Labor model (R3) that can create tensions and limitations for healthy living. Numerous studies in social epidemiology have sought to explain these influences [170–172].

Through relationship R4, other influences on the Local Human Environment, such as an atmosphere of pessimism, excess competition, or fear, can have repercussions on individual Mind System (R115). R4 also affects the Local Physical Environment, where most food outlets offer unhealthy food products. Finally, Society offers to Health Services Scientific and Technological Knowledge and Culture (R5), that adopt models emphasizing pharmacological control, and affecting the educational possibilities of the relation R106 (Cycle 1), and of the relation R107—Health Care Influence on the Human Environment (family).

The interactions between the Local Physical Environment and the Local Human Environment (R112) have a potential impact by creating and expanding factors that produce/maintain BP. For example, violence, originating from the Local Human Environment, can generate the destruction of public equipment or interdiction of public spaces that would otherwise favor physical exercise. Obviously, relationships R6 and R7, which define government actions, heavily influence Health Services.

## Closing Remarks

One of this study objectives–“to create an integrated, coherent, and increasingly precise conceptual framework, capable of aggregating different partial or specialized studies,”–was partially met by this study, which presents the first level of detail. The model advances scientific knowledge, to the extent that it integrates a large set of themes addressed in the literature, which generally display a narrow focuses and little dialogue between the various components that condition the prevalence and incidence of CVD&Diabetes in the contemporary world.

We can classify the literature related to Systems Science in health research in three large groups: paradigmatic and theoretical studies, standpoint and prescriptive studies, sometimes discussing potential uses, and application studies, mostly of simulations of system dynamics, agent-based modeling, and networks analysis [26]. Although these are the most used techniques, possibly due to their properties of quantification and formal validation, Van de Water, Schinkel & Rozier [173] present a survey and classification of the literature on “soft” systemic methods applied to health studies, which frequently require the exploration of questions on the nature of the knowledge itself and its premises, the “system paradigm”.

There are nuances in this “hard” versus “soft” categorization. Challenging the inevitability of excessively restricting and simplifying the phenomena in order to build quantitative systemic models, intermediate approaches have emerged [174]. Although essentially qualitative, they are conceptually and technically consistent and replace quantitative expression with the understanding of the overall dynamic structure, thereby allowing a better understanding of certain systemic behaviors that measurements do not reveal or cannot be obtained.

In the next stage of the model’s development, the various subsystems will be “zoomed” in their systemic structure, i. e, “sub-subsystems” and the relations between them. Of particular interest are the subsystems “Mind System”, “Active Life”, and “Body System”, the latter representing the physiological phenomena associated with the cardiovascular and metabolic processes of interest. By following the different flows of relationships at the two levels of decomposition of different subsystems, the objective is to reveal new associations between the various dimensions and scientific specialties. In the decomposition of the “Human Environment”, one must also consider the additional possibility of studying it through network techniques, examining its topology and its potential for multiplying or blocking behaviors and information flows, etc.

From the methodological perspective, our study, while offering a complex and rich “soft” view, open multiple possibilities of quantitative applications, thus adding to the possibilities for dealing with complexity in health. There are many possibilities for future methodological studies. One is “causal loop” diagrams derived from the model, which can shed more light on stability cycles in the system as a whole. For that one would define a macro-variable representing each subsystem’s overall state, and by following the relationships that connect these variables, evaluate how they affect each other, without needing to quantify them, but only indicating the tendency to increase or decrease by their interaction. The resulting diagram would deepen our understanding of the cycles of systemic stability and instability [175]. Another suggested path is the integration of quantitative methods, whether in statistical, analytic, or computational models, nested in the framework proposed here.

The other objective of the paper–“to orient planning of strategic actions […] pointing to new paths for intervention”–is an essential component of the approach proposed here, to the extent that: (i) it identifies the internal circularities and cycles that generate the system’s resistance to change; (ii) it allows discovering the necessary leverage points for the intervention’s success; and (iii) it orients the choice of adequate strategies through the creation of scenarios. We believe that this study can contribute to a revision of the research agenda and to orient integrated actions in the field of public health.
